# Detecting number processing and mental calculation in patients with disorders of consciousness using a hybrid brain-computer interface system

**DOI:** 10.1186/s12883-015-0521-z

**Published:** 2015-12-15

**Authors:** Yuanqing Li, Jiahui Pan, Yanbin He, Fei Wang, Steven Laureys, Qiuyou Xie, Ronghao Yu

**Affiliations:** 1 Center for Brain Computer Interfaces and Brain Information Processing, South China University of Technology, Guangzhou, 510640 China; 2 Guangzhou Key Laboratory of Brain Computer Interaction and Applications, Guangzhou, China; 3 School of Software, South China Normal University, Guangzhou, 510641 China; 4 Coma Research Group, Centre for Hyperbaric Oxygen and Neurorehabilitation, General Hospital of Guangzhou Military Command of People’s Liberation Army, Guangzhou, 510010 China; 5 Coma Science Group, Cyclotron Research Centre and Neurology Department, University and University Hospital of Liège, 4000 Liège, Belgium

**Keywords:** Number processing, Mental calculation, Disorders of consciousness, Brain computer interface, P300, Steady-state visual evoked potential

## Abstract

**Background:**

For patients with disorders of consciousness such as coma, a vegetative state or a minimally conscious state, one challenge is to detect and assess the residual cognitive functions in their brains. Number processing and mental calculation are important brain functions but are difficult to detect in patients with disorders of consciousness using motor response-based clinical assessment scales such as the Coma Recovery Scale-Revised due to the patients’ motor impairments and inability to provide sufficient motor responses for number- and calculation-based communication.

**Methods:**

In this study, we presented a hybrid brain-computer interface that combines P300 and steady state visual evoked potentials to detect number processing and mental calculation in Han Chinese patients with disorders of consciousness. Eleven patients with disorders of consciousness who were in a vegetative state (*n* = 6) or in a minimally conscious state (*n* = 3) or who emerged from a minimally conscious state (*n* = 2) participated in the brain-computer interface-based experiment. During the experiment, the patients with disorders of consciousness were instructed to perform three tasks, i.e., number recognition, number comparison, and mental calculation, including addition and subtraction. In each experimental trial, an arithmetic problem was first presented. Next, two number buttons, only one of which was the correct answer to the problem, flickered at different frequencies to evoke steady state visual evoked potentials, while the frames of the two buttons flashed in a random order to evoke P300 potentials. The patients needed to focus on the target number button (the correct answer). Finally, the brain-computer interface system detected P300 and steady state visual evoked potentials to determine the button to which the patients attended, further presenting the results as feedback.

**Results:**

Two of the six patients who were in a vegetative state, one of the three patients who were in a minimally conscious state, and the two patients that emerged from a minimally conscious state achieved accuracies significantly greater than the chance level. Furthermore, P300 potentials and steady state visual evoked potentials were observed in the electroencephalography signals from the five patients.

**Conclusions:**

Number processing and arithmetic abilities as well as command following were demonstrated in the five patients. Furthermore, our results suggested that through brain-computer interface systems, many cognitive experiments may be conducted in patients with disorders of consciousness, although they cannot provide sufficient behavioral responses.

## Background

Recently, significant attention has been paid to the exploration and assessment of residual cognitive functions in patients with disorders of consciousness (DOC), i.e., comas, vegetative states (VS), and minimally conscious states (MCS), which are among the most mysterious and least understood conditions of the human brain [1–3]. Currently, behavior-based methods are predominantly used in evaluating patients with DOC [4]. However, these patients usually lack the capacity for normal physical movements [5], which limits the application of behavior-based methods in many cases. For instance, clinical assessments based on scales such as the Glasgow Coma Scale (GCS) or the Coma Recovery Scale-Revised (CRS-R) rely on motor responses to external stimuli at the time of observation [6]. Consequently, clinical misdiagnosis rates are relatively high, ranging from 37 to 43 % in VS and MCS patients [2, 7]. Recently, neuroimaging techniques such as functional magnetic resonance imaging (fMRI), electroencephalography (EEG) and transcranial magnetic stimulation (TMS) have been proposed for use in probing residual brain function in certain patients with DOC [8, 9]. For instance, command-specific changes were detected in fMRI or EEG signals, and motor-independent evidence of awareness was observed in several studies [1, 2, 10–15]. Furthermore, an fMRI experiment was designed in which visual cognition that included the passive processing of light, color, motion, coherent shapes, and object categories was assessed in a patient with a severe DOC [3].

Number processing and mental calculation are important brain functions associated with other cognition functions, including symbol representation and operation, attention, working memory and linguistic processing [16]. To the best of our knowledge, number processing and mental calculation, which are expected to be impaired to a certain degree, have not been studied in patients with DOC. The neural basis of number processing has been studied using neuroimaging and other neuroscientific methods. For instance, findings based on fMRI as well as single-unit recordings in monkeys suggested preferential involvement of the bilateral intraparietal sulcus (IPS) and the medial parietal structures in approximation and of the angular gyri in exact calculation [17–21]. Many studies have also characterized deficits in calculation performance and related them to lesion sites in neurological patients [18]. For instance, loss of gray matter in the IPS has been observed in two medical conditions, prematurity [22] and Turner’s syndrome [23], which are associated with dyscalculia. Mental calculation abilities were found to be commonly impaired early in the course of Alzheimer’s disease (AD) [24, 25]. The numerical deficits observed in Parkinson’s disease and the functional relationship between numerical and other cognitive deficits were assessed in a study [26], the results of which suggested that impairments in both working memory and executive function lead to secondary deficits in numerical processing. Finally, calculation tasks have been included in the Montreal Cognitive Assessment (MoCA) and Mini-mental State Examination (MMSE), which are commonly used in the evaluation of the cognitive impairments observed in AD, stroke, and Parkinson’s disease [27–29]. However, clinical assessment scales such as the GCS and the CRS-R for patients with DOC do not contain number and calculation tasks. One possible reason is that in patients with DOC, severe motor response deficits or weak residual motor responses are insufficient to support behavioral experiments that involve number processing. By exploring number processing and mental calculation in patients with DOC, we might be able to evaluate their residual cognitive functions more widely and observe the extent to which the multiple brain functions associated with number processing and calculation are impaired after severe brain injury.

Brain-computer interfaces (BCIs) provide non-muscular communication and control by directly translating brain activities recorded from the scalp into computer control signals, thereby enabling users with motor disabilities to convey their intent to the external world [6, 30]. Therefore, BCIs may offer the potential to explore residual cognition functions in patients with DOC, including number processing and calculation. When BCIs are applied to patients with DOC, the online feedback may have positive effects that allow the conscious patients to effectively perform the instructed tasks, and feedback significantly above the level predicted by chance provides evidence of residual brain function to the examiners. Additionally, BCIs could serve as supportive tools for detecting consciousness in patients with DOC by detecting responses to commands and communications. Lule et al. tested a 4-choice auditory P300-based BCI on 13 MCS, 3 VS, and 2 LIS patients, among whom one LIS patient had a significant correct response rate of 60 % [4]. Coyle et al. used a motor imagery-based BCI to detect awareness in four MCS patients and to determine whether these patients may learn to modulate sensorimotor rhythms with visual or auditory feedback. The results indicated that all four patients achieved accuracies above the 70 % criterion level for a 2-class BCI in multiple sessions and thus had the capacity to operate a simple BCI-based communication system with real-time visual and auditory feedback [14].

Recent studies have validated the effectiveness of hybrid BCIs, which directly combine two or more different types of brain signals such as P300 potentials and steady state visual evoked potentials (SSVEPs) [31]. In our previous study [32], a hybrid BCI for brain switch application was developed in which P300 and SSVEPs were combined to improve target detection performance. Experimental results obtained from the healthy subjects showed that the target detection performance was better for the hybrid BCI than for the P300- or SSVEP-based BCI.

Considering the above-mentioned factors, here, we propose a visual hybrid BCI that combines P300 potentials and SSVEPs (a variant of our previously established system) [32] for detecting the number and calculation abilities of patients with DOC. The patients’ real-time answers to arithmetic problems were presented via BCI feedback. Eleven patients participated in the experiment, five of whom achieved accuracies significantly higher than the chance level. Command following and number and arithmetic abilities were thus demonstrated in these patients. Additionally, this study showed that by applying the BCIs, we could conduct cognitive experiments for patients with DOC, even if they are unable to demonstrate sufficient behavioral responses in these experiments.

## Methods

### Patients

This study was undertaken at the General Hospital of Guangzhou Military Command of People’s Liberation Army in Guangzhou, China, between July and December 2013. Brain activity was detected only if the patients were free of centrally acting sedative drugs. Eleven brain-injured Han Chinese patients participated in this experiment (5 males; 6 VS, 3 MCS and 2 EMCS [emerged from MCS]; mean age ± standard deviation (SD), 38.55 ± 11.98 years; see Table 1). No patient had a history of impaired visual acuity before brain injury. Additionally, four healthy subjects (HC1, HC2, HC3, and HC4) with no history of neurological disease (three males; mean age ± SD, 29 ± 2 years) were included in our experiment as a control group. The experiments for patients with DOC and healthy subjects were approved by the Ethical Committee of the General Hospital of Guangzhou Military Command of People’s Liberation Army in Guangzhou, which complies with the Code of Ethics of the World Medical Association (Declaration of Helsinki). Written informed consent was obtained from the patients’ legal surrogates and the healthy subjects for the experiments and publication of their individual details in this manuscript. The clinical diagnoses were based on the CRS-R, which is composed of six subscales that address auditory, visual, motor, oromotor, communication and arousal functions [33]. Scoring on each subscale is based on the presence or absence of operationally defined behavioral responses to specific sensory stimuli. For the purpose of the experiment, the eleven patients attended two CRS-R assessments, one in the week before the experiment and the other in the week after the experiment. The CRS-R scores for each patient are presented in Table 1. The VS is characterized by the return of arousal without recovery of awareness. The MCS is defined by the presence of inconsistent but reproducible goal-directed behaviors (e.g., response to command, visual pursuit, and localization of noxious stimulation). The EMCS is characterized by reliable and consistent demonstration of functional interactive communication or functional use of two different objects. Note that certain patients who have an amount of residual cognitive functions (e.g., command following) may meet the behavioral criteria for VS because they cannot provide sufficient behavioral responses during the clinical diagnoses based on the CRS-R [13, 34].

**Table 1:** Summary of patient clinical status. The clinical diagnosis states in the brackets were obtained after the experiment

Patient	Clinical diagnosis		CRS-R score (sub-scores)	
			Before the experiment	After the experiment
VS1	VS	(VS)	6 (1-0–2–1–0–2)	6 (1–0–2–1–0–2)
VS2	VS	(EMCS)	7 (1–1–2–1–0–2)	17 (4–4–5–1–1–2)
VS3	VS	(VS)	4 (1–1–0–0–0–2)	6 (1–0–2–1–0–2)
VS4	VS	(EMCS)	3 (0–0–1–0–0–2)	23 (4–5–6–3–2–3)
VS5	VS	(MCS)	6 (1–0–2–1–0–2)	10 (2–0–4–2–0–2)
VS6	VS	(VS)	5 (1–0–1–1–0–2)	6 (1–0–2–1–0–2)
MCS1	MCS	(EMCS)	10 (1–3–3–1–0–2)	19 (3–5–6–1–1–3)
MCS2	MCS	(MCS)	8 (1–2–2–1–0–2)	9 (1–2–3–1–0–2)
MCS3	MCS	(MCS)	8 (1–2–2–1–0–2)	9 (1–3–2–1–0–2)
EMCS1	EMCS	(EMCS)	16 (1–3–5–3–2–2)	23 (4–5–6–3–2–3)
EMCS2	EMCS	(EMCS)	14 (1–3–3–3–2–2)	20 (4–5–3–3–2–3)

Note: Coma Recovery Scale–Revised subscales: auditory, visual, motor, oromotor, communication, and arousal functions

### Data acquisition

A NuAmps device (Neuroscan, Compumedics Ltd, Victoria, Australia) was used to collect scalp EEG signals. Each patient wore an EEG cap (LT 37) with Ag-AgCl electrodes. The EEG signals were referenced to the right mastoid. According to the standard 10–20 system [35], the EEG signals used for analysis were recorded from 10 electrodes: “Fz”, “Cz”, “P7”, “P3”, “Pz”, “P4”, “P8”, “O1”, “Oz” and “O2” [32]. The EEG signals were amplified, sampled at 250 Hz and band-pass filtered between 0.1 Hz and 30 Hz.

### BCI paradigm

**Fig. 1:**
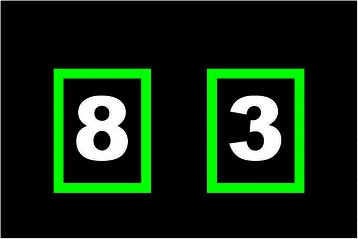
GUI of the hybrid BCI, in which two buttons portraying single-digit numbers are randomly displayed on the left and right sides in each trial. The left and right number buttons, one of which showed the answer to an arithmetic problem given to the patients, flickered from appearance to disappearance on a black background at frequencies of 6.0 Hz and 7.5 Hz, respectively. Simultaneously, the two button frames flashed in and out of view in a random order

The Graphical User Interface (GUI) used in this study is illustrated in Fig. 1. Two buttons displaying single-digit Arabic numbers, one of which was the answer to the arithmetic problem given to the patients in our experiment, were displayed on the left and right sides of the GUI. Each button (size: 6.6 cm × 9 cm) was placed in the center of a green button frame (size: 8.6 cm × 11 cm, margin width: 1 cm). The horizontal distance between the two button frames was 4 cm in the GUI. The area ratio of the button, the button frame and the GUI was 0.07:0.1:1.

The two number buttons on the left and right sides of the GUI flickered from appearance to disappearance at 6.0 Hz and 7.5 Hz, respectively. Simultaneously, the two button frames flashed from appearance to disappearance in a random order, with each appearance lasting 200 ms and an 800-ms interval between two consecutive appearances. The patients were instructed to focus on the target number button (the answer to an arithmetic problem) and to count the number of flashes of the corresponding button frame. In this manner, the SSVEP and P300 responses could be simultaneously elicited by the flickering target number button and the flashing button frame, respectively.

### Experimental design and procedure

During the experiment, each patient was seated in a comfortable wheelchair approximately 0.5 m from a 22-in LED monitor. A preliminary screening was conducted before the experiment to explain the experimental procedure to the patient.

Before the online evaluation of the first block, each patient performed a calibration run of 10 trials using the GUI shown in Fig. 1. Specifically, in each trial, the left and right buttons flickered for 10 s at 6.0 Hz and 7.5 Hz, respectively. Simultaneously, the two button frames flashed in a random order, with each button frame flashing five times. The patient was instructed to pay attention to the target number button and to count the flashes of the corresponding button frame. We trained an initial Support Vector Machine (SVM) classifier for P300 detection using EEG data from the calibration run. Furthermore, the P300 classification model was updated after each block of online evaluation using the data from the last two blocks (20 trials). For example, after Block 4, we used the data from Blocks 3 and 4 to update the model for the online classification in Block 5. The rationale for training the P300 model in this manner was threefold: first, because the patients were easily fatigued, the calibration procedure needed to be as short as possible, and performing a new calibration run before each evaluation block on a separate day was not appropriate; second, due to the fluctuating state of the DOC patients, we could not use a fixed model during the experiment, which lasted a long time (e.g., more than 1 month); third, to partially overcome these problems, we used a semi-supervised learning approach for addressing a small training dataset based on previous studies [36–38]. Specifically, we used the data from the latest trials to update the SVM model. Note that the results of healthy subjects in this study demonstrated the effectiveness of this method for updating the SVM model.

**Fig. 2:**
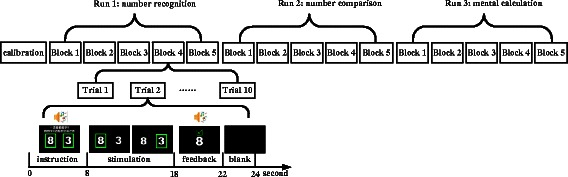
Experimental paradigm. Five blocks were included for each run, and each block consisted of 10 trials. Each trial began with a visual and auditory presentation of the task instructions. Next, two number buttons were randomly displayed on the left and right sides of the GUI. After the instruction, the two number buttons flickered, and the two corresponding button frames were highlighted. After 10 s, auditory (i.e., applause) and visual feedback (i.e., number selected by the classification algorithm) were presented

In the online evaluation, three experimental runs were conducted for each patient. Figure 2 illustrates the online experimental paradigm. Each patient was instructed to perform number recognition, number comparison and mental calculation (single-digit addition and subtraction) in Runs 1, 2 and 3, respectively, while EEG data were collected and processed online. Each run contained five blocks, and each block was composed of 10 trials. Different blocks were conducted on separate days because the patients were easily fatigued. Each subject performed at most two blocks per week, and each subject accomplished 3 runs of the experiment in approximately 2 months. Note that the tasks of Runs 1, 2, and 3 became increasingly more difficult for the patient. Using these three runs, we could evaluate the capacity for numerical/symbolic processing in patients with DOC.

Each trial began with visual and auditory presentation of the task instructions in Chinese, which lasted 8 s. The instructions were “Focus on the target number (e.g., 8) and count the flashes during which the target number frame is highlighted” for Run 1 and “Focus on the larger/smaller number and count the flashes during which the larger/smaller number frame is highlighted” for Run 2. For Run 3, a predefined addition/subtraction problem (e.g., “3 + 5 = ?”) was first presented visually and aurally. Next, the instruction “Focus on the correct answer and count the flashes during which the correct answer frame is highlighted” was presented. Simultaneously, in these three runs, two different numbers were randomly displayed on the left and right sides of the GUI. More specifically, the two numbers were randomly chosen from the single-digit numbers 1 to 9 for Runs 1 and 2 (one of which was used as the target for Run 1). For Run 3, one of the two numbers was the correct answer, and the other was randomly chosen from the single-digit numbers 1 to 9. A total of 25 trials were presented for finding the larger number/addition calculation; 25 trials, for finding the smaller number/subtraction calculation for Run 2/Run 3. After the instruction was presented, the two buttons flickered while the two corresponding button frames flashed, as in the calibration run. The patients were asked to focus on the target number button and count the number of flashes of the corresponding button frame. After 10 s, a feedback number, which was determined by the BCI algorithm, appeared in the center of the GUI. If the result was correct, a tick and an audio clip of applause were presented for 4 s to encourage the patient. Otherwise, a cross was displayed for 4 s. A break of at least 10 s occurred between two consecutive trials, depending on the patient’s level of fatigue. During the experiment, the patient was carefully observed by an experienced doctor to ensure task engagement. A trial was discarded if the patient showed decreased arousal (i.e., closed his/her eyes) or continuous body movements (e.g., resulting in a cough) for more than 5 s. The next trial was started after the patient showed a prolonged period of spontaneous eye opening or reawakening.

### Data analysis

The procedures for P300 and SSVEP detection have been described previously [32]. In brief, the P300 and SSVEP detectors were separately designed, and the EEG data were simultaneously fed into the two detectors.

### P300 detection

First, the EEG signals were filtered between 0.1 Hz and 10 Hz. For each flash of a button frame, we extracted a segment of the EEG signal from each channel (0 to 800 ms after intensification of the button frame). This segment was down-sampled by a rate of 5 to obtain a data vector (with a length of 40). We concatenated the vectors from all 10 channels to obtain a new data vector corresponding to the button frame flash. Next, we constructed a features vector for each button frame by averaging the data vectors across the five flashes in a trial. All of the feature vectors were normalized by mapping them into the range [−1, 1]. Finally, the SVM classifier was applied to the two feature vectors corresponding to the two buttons, and two SVM scores were obtained for each trial.

### SSVEP detection

The EEG signals were first filtered between 4 Hz and 10 Hz. After the stimulus onset, we extracted segments of 10-s EEG signals (2500 data points) from the following eight channels: “P7”, “P3”, “Pz”, “P4”, “P8”, “O1”, “Oz” and “O2” [39]. For each segment, we used the minimum energy combination (MEC) to combine the signals from multiple channels [40]. Specifically, the signal vector **s** is defined as the weighted combination of the channel signals.

1 $$ \mathbf{s}={\displaystyle \sum_{i=1}^{N_y}{w}_i{\mathbf{y}}_{\mathbf{i}}}=\mathbf{Y}{\mathbf{w}}^T, $$

where *N*_*y*_ is the number of channels (*N*_*y*_ = 8 in this study), $\mathbf{Y}=\left[{\mathbf{y}}_{\mathbf{1}}, \dots, {\mathbf{y}}_{{\mathbf{N}}_{\mathbf{y}}}\right],$ where *y*_*i*_ is the *i*th channel signal, and $\mathbf{w}=\left[{\mathrm{w}}_1, \dots, {\mathrm{w}}_{{\mathrm{N}}_{\mathrm{y}}}\ \right]$ is a weight vector.

To obtain the weight vector, an orthogonal projection is used to remove any potential SSVEP activity from the recorded signal.

2 $$ \overline{\mathbf{Y}}=\mathbf{Y}-\mathbf{X}{\left({\mathbf{X}}^T\mathbf{X}\right)}^{-1}{\mathbf{X}}^T\mathbf{Y}, $$

where **X** is the SSVEP information matrix that contains the sine and cosine components associated with the harmonics of the flickering frequency, and $\overline{\mathbf{Y}}$ is the remaining signal matrix that contains noise, artifact, and background brain activity.

Next, the weight vector **ŵ** is found by minimizing the energy of the signal $\overline{\mathbf{Y}}$

3 $$ \underset{\hat{\mathbf{w}}}{ \min }{\left\Vert \overline{\mathbf{Y}}{\widehat{\mathbf{w}}}^T\right\Vert}^2=\underset{\widehat{\mathbf{w}}}{ \min}\widehat{\mathbf{w}}{\overline{\mathbf{Y}}}^T\overline{\mathbf{Y}}{\widehat{\mathbf{w}}}^T. $$

By solving the optimization problem (3), *N*_*y*_ weight vectors denoted as ${\mathbf{w}}^{{}_{\mathbf{1}}}, \dots, {\mathbf{w}}^{{}_{{\mathbf{N}}_{\mathbf{y}}}}$ are obtained based on the eigenvalues $\left({\lambda}_1, \dots, {\lambda}_{N_y}\right)$ in ascending order and the corresponding eigenvectors $\left({\boldsymbol{\upnu}}_{\mathbf{1}}, \dots, {\boldsymbol{\upnu}}_{{\mathbf{N}}_{\mathbf{y}}}\right)$ as follows:

4 $$ W=\left[{\mathbf{w}}^{{}_{\mathbf{1}}}, \dots, {\mathbf{w}}^{{}_{{\mathbf{N}}_{\mathbf{y}}}}\right]=\left[\frac{{\mathbf{v}}_{\mathbf{1}}}{\sqrt{\lambda_1}}, \dots, \frac{{\mathbf{v}}_{{\mathbf{N}}_{\mathbf{y}}}}{\sqrt{\lambda_{N_y}}}\right] $$

Furthermore, *N*_*y*_ new signals are created using these weight vectors, as in (1). We select the first $\overline{N_y}$ of the newly defined *N*_*y*_ signals that satisfies

5 $$ {\displaystyle \sum_{i=1}^{\overline{N_y}}{\lambda}_i}/{\displaystyle \sum_{j=1}^{N_y}{\lambda}_i}>0.1 $$

This approach can be interpreted as selecting the number of channels to discard approximately 90 % of the nuisance signal energy [40].

SSVEP detection is based on the $\overline{N_y}$ signals obtained as described above. Using a discrete Fourier transformation, the normalized power density spectrum for the *j*th signal vector of the $\overline{N_y}$ signals is calculated by

6 $$ P\left(j,f\right)=\frac{\mathrm{FFT}\left({\overline{y}}_j\right)}{{\displaystyle \sum \left|\mathrm{F}\mathrm{F}\mathrm{T}\left({\overline{y}}_j\right)\right|}}, $$

where *f* represents frequency, ${\overline{y}}_j$ is the *j*th signal vector of the $\overline{N_y}$ signals, and $\mathrm{F}\mathrm{F}\mathrm{T}\left({\overline{y}}_j\right)$ is the fast Fourier transform of ${\overline{y}}_j$. Furthermore, ${\displaystyle \sum \left|\mathrm{F}\mathrm{F}\mathrm{T}\left({\overline{y}}_j\right)\right|}$ denotes summation over the total frequency points of the spectrum; therefore, the sum of the power density spectrum is normalized to one [41].

During the Fourier analysis, we used the zero padding method to increase the number of data points from 2500 to 4096 (a power of 2). In this case, the frequency resolution is 0.061 Hz. We further integrated the power

7 $$ \overset{\wedge }{P}(f)=\frac{1}{\overline{N_y}{N}_h}{\displaystyle \sum_{j=1}^{\overline{N_y}}{\displaystyle \sum_{k=1}^{N_h}P\left(j,kf\right)}}, $$

where *N*_*h*_ is the number of harmonics taken into account (*N*_*h*_ = 2 in this study). Thus, the power of the SSVEP response for the *i*th number is $\overset{\wedge }{P}\left({f}_i\right),$, where *f*_*i*_ is the stimulation frequency of the flickering number *i*.

Furthermore, for each flickering number, we calculated the ratio of the mean power in a narrow band (band width: 0.1 Hz) to that in a wide band (band width: 1 Hz). For each trial, two power ratios were obtained for the two buttons with different flickering frequencies.

### Decision making

For each trial, we summed the SVM score for P300 detection and the power ratio of SSVEP detection for each number button and chose the number button with the higher summed value as the feedback result.

### Performance evaluation

For each subject and each run, the accuracy rate was calculated as the ratio of the number of all correct responses (hits) to the total number of trials. To assess the significance of the accuracy, we calculated the *χ*^2^ statistic as shown below [42]:

8 $$ {\chi}^2={\displaystyle \sum_{i=1}^2\frac{{\left(f{o}_i-f{e}_i\right)}^2}{f{e}_i}}, $$

where *fo*_1_ or *fo*_2_ is the observed number of hits or misses, and *fe*_1_ or *fe*_2_ represents the expected number of hits and misses, respectively. The degree of freedom was 1 in our experiment with two possible choices for each trial. Considering that each run of our experiment had 50 trials, 25 hits and 25 misses could be expected by chance. Using a significance level of *p* = 0.05, we obtained a value of 3.84 for *χ*^2^ (degree of freedom: 1), which corresponded to 32 hits in 50 trials or an accuracy of 64 %.

In this study, we also used approximate entropy (ApEn) to assess the level of consciousness in patients with DOC. EEG-based ApEn has been shown to correlate to changes in conscious state [43–46]. The ApEn of a time series {*u*(*i*)} can be computed as follows. First, a sequence of *m* - dimension vectors *X*(*i*) is constructed:

9 $$ X(i)=\left[u(i),u\left(i+1\right),\dots, u\left(i+m-1\right)\right], $$

where *i* = 1, 2, …, *N* − *m* + 1, and *N* is the data length (2500 EEG data points in each trial in this study). The distance between two vectors is defined as the maximum absolute difference between the corresponding elements, i.e.,

10 $$ d\left[X(i),X(j)\right]= \max \left\{\left|u\left(i+k\right)-u\left(j+k\right)\right|,\ 0\le \mathrm{k}\le \mathrm{m}\hbox{-} 1\right\}, $$

Next, for each *X*(*i*), the relative number of vectors *X*(*j*) satisfying *d*[*X*(*i*), *X*(*j*)] ≤ *r* is calculated and denoted as *N*^*m*^(*i*), where *r* is the tolerance value. Finally, the ApEn is obtained as follows:

11 $$ ApEn\left(m,r\right)={\phi}^m(r)-{\phi}^{m+1}(r), $$

12 $$ {\phi}^m(r)=\frac{1}{N-m+1}{\displaystyle \sum_{i=1}^{N-m+1} ln\frac{N^m(i)}{N-m+1}}, $$

In this study, we set *m* = 2 and *r* = 0.25 × *SD* (standard deviation of 2500 EEG data points), which could produce reasonable statistical validity for ApEn [47]. For each trial, ApEn was computed using the “Pz” electrode, according to the related references [43–46].

## Results

Table 2 summarizes the accuracy rates of the online experiment. In Run 1, five of the eleven patients (patients VS2, VS4, MCS1, EMCS1 and EMCS2) achieved accuracies greater than 64 % (ranging from 66 to 80 %), corresponding to significance levels of less than 0.05. In Run 2, five patients (patients VS2, VS4, MCS1, EMCS1 and EMCS2) achieved accuracies greater than 64 % (ranging from 66 to 72 %). In Run 3, three of the five patients (patients VS2, MCS1 and EMCS1) achieved accuracies greater than 64 % (ranging from 64 to 66 %). For patients VS1, VS3, VS5, VS6, MCS2 and MCS3, the accuracies were not significant in Runs 1, 2 or 3. During the experimental period, patients VS4 and EMCS1 began to speak, and patient VS1 left the hospital for economic reasons. These three patients thus performed only Runs 1 and 2. Furthermore, four healthy subjects (HC1, HC2, HC3, and HC4) achieved accuracies higher than the significance level of 64 % (ranging from 88 to 100 %) in Runs 1, 2, and 3.

**Table 2:** Online accuracy rates for the subjects

Subject	Run 1 (number recognition)		Run 2 (number comparison)		Run 3 (addition and subtraction)		Average accuracy
	Number of trials	Accuracy (*p*-value)	Number of trials	Accuracy (*p*-value)	Number of trials	Accuracy (*p*-value)	
VS1	50	60 % (0.157)	50	52 % (0.777)			56 %
	*51*	*58.8 % (0.208)*					*55.4 %*
VS2	50	**66 % (0.024)**	50	**66 % (0.024)**	50	**64 % (0.048)**	**65.3 %**
			*52*	***65.4 % (0.027)***	51	***64.7 % (0.036)***	***65.4 %***
VS3	50	58 % (0.258)	50	58 % (0.258)	50	48 % (0.777)	54.7 %
					*52*	*50 % (1.000)*	*55.3 %*
VS4	50	**70 % (0.005)**	50	**66 % (0.024)**			**68 %**
			*55*	***63.6 % (0.043)***			***66.7 %***
VS5	50	56 % (0.396)	50	60 % (0.157)	50	48 % (0.777)	54.7 %
	*51*	*56.9 % (0.327)*					*55 %*
VS6	50	56 % (0.396)	50	52 % (0.777)	50	52 % (0.777)	53.3 %
			*51*	*52.9 % (0.674)*			*54 %*
MCS1	50	**66 % (0.024)**	50	**72 % (0.002)**	50	**64 % (0.048)**	**67.3 %**
			*56*	***67.9 % (0.008)***			***66 %***
MCS2	50	62 % (0.090)	50	52 % (0.777)	50	46 % (0.572)	53.3 %
	*51*	*62.7 % (0.069)*					*53.6 %*
MCS3	50	60 % (0.157)	50	56 % (0.396)	50	58 % (0.258)	58 %
	*53*	*56.6 % (0.216)*					*58.6 %*
EMCS1	50	**80 % (<0.001)**	50	**72 % (0.002)**			**76 %**
EMCS2	50	**76 % (<0.001)**	50	**70 % (0.005)**	50	**66 % (0.024)**	**70.7 %**
	*54*	***74.1 % (<0.001)***			*54*	***64.8 % (0.030)***	***69.6 %***
HC1	50	**88 % (<0.001)**	50	**92 % (<0.001)**	50	**94 % (<0.001)**	**91.3 %**
HC2	50	**94 % (<0.001)**	50	**92 % (<0.001)**	50	**96 % (<0.001)**	**94 %**
HC3	50	**98 % (<0.001)**	50	**100 % (<0.001)**	50	**96 % (<0.001)**	**98 %**
HC4	50	**100 % (<0.001)**	50	**92 % (<0.001)**	50	**94 % (<0.001)**	**95.3 %**

Note: For each patient two rows of results are provided. The results (in italics) in the second row were obtained based on the total number of trials (i.e., including the rejected trials), where the empty table cells indicated that no trial was discarded in this run. For the four healthy subjects, no trials were discarded. Accuracies with significance (*p* < 0.05) are highlighted in bold

Table 3 summarizes the ApEn values for the 11 patients and four healthy subjects. Five patients (VS2, VS4, MCS1, EMCS1 and EMCS2) who obtained accuracies higher than 64 % in Runs 1, 2 or 3 showed a mean ApEn value of 1.074 ± 0.251. The other patients with accuracies lower than 64 % showed a mean ApEn value of 0.684 ± 0.227. A significant difference of ApEn values existed between v’ (non-paired Student’s *t*-test, *p* < 0.001). Furthermore, four healthy subjects (HC1, HC2, HC3, and HC4) showed a mean ApEn value of 1.011 ± 0.106. No significant difference in the ApEn values was observed between the group of five patients (VS2, VS4, MCS1, EMCS1 and EMCS2) and the healthy group (non-paired Student’s *t*-test, *p* = 0.431).

**Table 3:** Mean approximate entropy values for the subjects

Subject	ApEn values (±SD)			
	Run 1 (number recognition)	Run 2 (number comparison)	Run 3 (addition and subtraction)	Average
VS1	1.121 (±0.500)	0.876 (±0.464)		0.998 (±0.473)
VS3	0.599 (±0.112)	0.558 (±0.178)	0.642 (±0.129)	0.600 (±0.137)
VS5	0.467 (±0.392)	0.514 (±0.143)	0.703 (±0.320)	0.561 (±0.300)
VS6	0.271 (±0.090)	0.618 (±0.432)	0.456 (±0.312)	0.475 (±0.339)
MCS2	0.966 (±0.332)	0.833 (±0.309)	0.475 (±0.093)	0.758 (±0.328)
MCS3	1.073 (±0.228)	0.788 (±0.151)	0.598 (±0.246)	0.820 (±0.282)
Average	0.684 (±0.227)			
VS2	1.152 (±0.319)	1.121 (±0.324)	1.140 (±0.327)	1.167 (±0.301)
VS4	1.021 (±0.363)	1.085 (±0.299)		1.053 (±0.315)
MCS1	0.865 (±0.207)	0.765 (±0.245)	0.870 (±0.278)	0.833 (±0.232)
EMCS1	0.800 (±0.154)	0.862 (±0.206)		0.831 (±0.175)
EMCS2	1.247 (±0.453)	1.625 (±0.143)	1.403 (±0.226)	1.425 (±0.324)
Average	1.074 (±0.251)			
HC1	1.077 (±0.247)	0.904 (±0.462)	1.173 (±0.324)	1.051 (±0.361)
HC2	0.881 (±0.156)	1.065 (±0.117)	0.890 (±0.262)	0.974 (±0.153)
HC3	1.191 (±0.229)	1.028 (±0.246)	0.911 (±0.212)	1.043 (±0.251)
HC4	1.022 (±0.280)	1.035 (±0.209)	0.951 (±0.300)	0.999 (±0.262)
Average	1.011 (±0.106)			

**Fig. 3:**
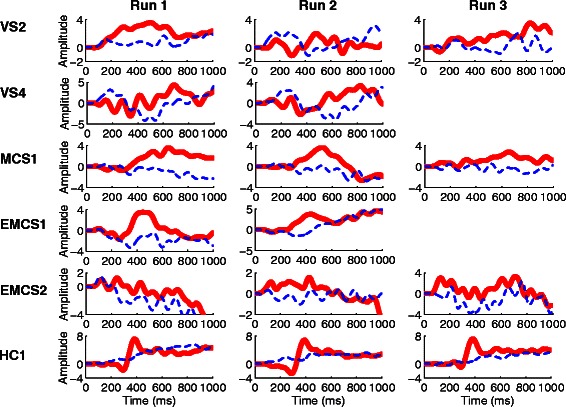
Grand-average P300 ERP waveforms from the “Pz” electrode in Run 1 (left panels), Run 2 (middle panels) and Run 3 (right panels) for five patients (VS2, VS4, MCS1, EMCS1, and EMCS2) and a healthy control. The red curves corresponding to target button frames contained P300 responses, whereas the blue curves corresponding to non-target button frames did not

For a healthy control (HC1) and the five patients (VS2, VS4, MCS1, EMCS1 and EMCS2) with significant accuracies, ERP waveforms from 0 ms to 1000 ms after stimuli onset were obtained by averaging the EEG channel signal across 50 trials in Run 1, 2 or 3. Figure 3 shows the average EEG signal amplitudes of the “Pz” electrode for the five patients and the healthy control. A P300-like component is apparent in all target curves.

**Fig. 4:**
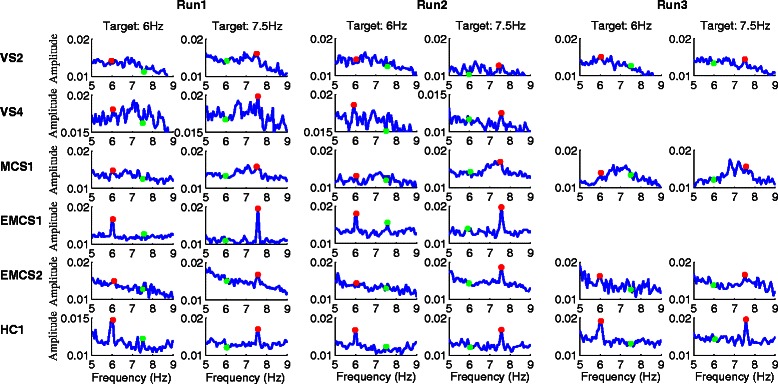
Average power density spectra of EEG signals from the eight selected electrodes (“P7”, “P3”, “Pz”, “P4”, “P8”, “O1”, “Oz” and “O2”) in all trials of Run 1 (left panels), Run 2 (middle panels) and Run 3 (right panels) for five patients (VS2, VS4, MCS1, EMCS1 and EMCS2) and a healthy control. The red and green points indicate flickering frequencies of the target and non-target buttons, respectively. The target frequencies of 6 Hz and 7.5 Hz imply that the target buttons appeared on the left and right sides of the GUI, respectively

For Runs 1, 2 and 3 and each subject (VS2, VS4, MCS1, EMCS1, EMCS2, and HC1), we obtained two average power density spectrum curves for the EEG signals across all trials with target buttons appearing at the left or right of the GUI, as shown in Fig. 4. The power spectrum for each trial was calculated using 10 s of the EEG signal from the eight selected channels (“P7”, “P3”, “Pz”, “P4”, “P8”, “O1”, “Oz” and “O2”), as in the SSVEP detection. Figure 4 shows that SSVEP was evoked at the target frequencies in most cases for the five patients and one healthy subject. Specifically, obvious SSVEP responses appeared in Run 1 for all five patients, in Run 2 for the patients VS4, MCS1, EMCS1, and EMCS2 (at the target frequency 7.5Hz), and in Run 3 for the patients VS2 and EMCS2 (note: the patients VS4 and EMCS1 performed only Runs 1 and 2), whereas the SSVEP responses were apparently evoked in all three runs for the healthy subject.

Furthermore, to determine whether the P300 or SSVEP aspect was more effective, the offline accuracies based on the P300 or SSVEP component for all patients and healthy controls were calculated using the data collected in Runs 1, 2, and 3 in a manner similar to that used in the online P300 or SSVEP detection. The results are listed in Table 4. For all runs for the 11 patients, no significant difference in accuracy was observed between the P300 and SSVEP components (*p* = 0.1638, paired Student’s *t*-test). However, Tables 2 and 4 show that for the five patients with significant accuracies, the online accuracy rates obtained by the hybrid features were significantly higher than those based on the P300 (*p* = 0.0027, paired Student’s *t*-test) or SSVEP component (*p* = 0.0028, paired Student’s *t*-test).

**Table 4:** Offline accuracies of individual P300 and steady-state visual evoked potential components for the patients

Patient	Offline accuracy rates					
	Run 1 (number recognition, 50 trials)		Run 2 (number comparison, 50 trials)		Run 3 (addition and subtraction, 50 trials)	
	P300	SSVEP	P300	SSVEP	P300	SSVEP
VS1	54 %	60 %	56 %	54 %		
VS2	60 %	**68 %**	60 %	58 %	**64 %**	60 %
VS3	60 %	50 %	56 %	46 %	50 %	46 %
VS4	62 %	**70 %**	60 %	62 %		
VS5	54 %	50 %	56 %	52 %	46 %	48 %
VS6	54 %	56 %	54 %	46 %	46 %	52 %
MCS1	**66 %**	60 %	**68 %**	**68 %**	58 %	62 %
MCS2	58 %	60 %	52 %	48 %	46 %	48 %
MCS3	58 %	58 %	54 %	50 %	54 %	54 %
EMCS1	**82 %**	**80 %**	**72 %**	**70 %**		
EMCS2	**72 %**	**64 %**	**68 %**	**64 %**	**64 %**	62 %
HC1	**84 %**	**80 %**	**88 %**	**82 %**	**90 %**	**90 %**
HC2	**92 %**	**90 %**	**88 %**	**86 %**	**92 %**	**90 %**
HC3	**96 %**	**94 %**	**98 %**	**96 %**	**90 %**	**92 %**
HC4	**98 %**	**92 %**	**88 %**	**88 %**	**94 %**	**88 %**

Note: Accuracies with significance (≥64 %, *p* < 0.05) are highlighted in bold

Among the six patients who were determined to be entirely vegetative based on repeated behavioral assessments, two patients (VS2 and VS4) progressed to MCS during the experiment. Furthermore, they subsequently emerged from MCS after the experiment. More interestingly, according to the CRS-R, five patients (patients VS2, VS4, MCS1, EMCS1 and EMCS2) who showed significant number processing and calculation ability improved their consciousness levels to a large degree. Table 1 shows the CRS-R scores for each patient before and after the experiment.

## Discussion

Detection of residual cognitive function and consciousness in patients who survive severe brain injury is highly challenging but crucial for accurate diagnosis, optimal care strategies and general quality of life. In this study, we used a hybrid BCI to explore number processing and calculation ability in patients with DOC. Our experiment consisted of three tasks: number recognition, number comparison and mental calculation. Eleven patients participated in our experiment, and five of these patients (2 VS, 1 MCS and 2 EMCS patients) showed significant number processing and calculation abilities (Table 2). ApEn measures the complexity (or irregularity) of a signal (Pincus, 1991). A larger ApEn value indicates higher irregularity, whereas a smaller ApEn value implies a more regular signal [48]. Table 3 shows the significant difference in ApEn between the five patients who obtained accuracy rates that were significantly higher than the chance level (50 %) and the other patients (*p* < 0.05). This finding indicated that the two groups of patients were in different conscious states [43–46]. No significant differences in the ApEn values were observed between the group of five patients and the healthy group (*p* = 0.431). Furthermore, clinical assessments based on the CRS-R showed that during the experimental period, the 5 patients recovered their levels of consciousness to a large degree (Table 1).

In addition to number processing and calculation ability, our experimental results also demonstrated command following ability in the five patients who obtained accuracies that were significantly higher than chance. First, the ability to follow commands was necessary for the 5 patients to effectively perform the tasks in our experiment. This finding indicates that the 5 patients paid attention to the target stimuli according to the instructions. An important observation is that significant differences in the evoked potential (P300 or SSVEP) exist between the patients and the healthy controls. For instance, the latent period of P300 was generally longer for the patients than for the healthy controls (Fig. 3), as has been reported in other studies [49, 50]. Furthermore, in several patients, the SSVEP was not apparently evoked as in the healthy controls (Fig. 4). In particular, distinguishing the amplitude of peaks driven by visual stimulation (i.e., target frequency 6 Hz or 7.5 Hz) and the amplitudes of non-task related peaks was difficult in Run 2 for patients VS2 and EMCS2 (at the target frequency 6 Hz) and in Run 3 for patient MCS1. However, P300 was elicited in these cases (Fig. 3). The main reason might be the fluctuating level of consciousness in patients with DOC over time, as low levels of consciousness impeded the efficient elicitation of SSVEP. Another reason might be that these patients might not simultaneously attend to the P300 and SSVEP stimuli as patient EMCS 1 and the healthy controls did, and neglecting the P300 or SSVEP stimuli might have allowed the corresponding evocation to deteriorate [51–53].

The human brain has remarkable capabilities for encoding and manipulating information related to quantities. Understanding how the brain processes numbers and quantities is a problem that is not only important for numerical cognition itself but is also relevant to understanding development, symbol representation and operation, action, memory, vision, language, executive function and cortical organization [54]. Studies on the neural basis of human number skill have suggested that the IPS, which can be activated in all number tasks, is the locus of core numerical processing. Areas of the pre-central and inferior prefrontal cortex are also activated when subjects are engaged in mental calculation [55]. Furthermore, two distinct circuits have been identified for arithmetic: the bilateral IPS for tasks involving the explicit representation of magnitude and the angular gyrus for the retrieval of previously learned facts [54, 56, 57]. Pathologies of these systems may lead to impairments of number processing in the brain (e.g., acalculia in adults and developmental dyscalculia in children). For patients with brain damage, assessing number and calculation ability is of substantial importance. First, numbers and calculation are important aspects of the cognition function of the human brain. Second, to a certain degree, we may understand other cognitive functions (i.e., language and executive function) in these patients with DOC through the assessment of number and calculation ability. Several scales, including the MOCA and MMSE, contain mental calculation-based indices that are commonly used to evaluate mental states for patients with AD, stroke and Parkinson disease, among others [27, 29]. For patients with DOC, clinical assessment scales such as the GCS and the CRS-R do not contain number and calculation tasks. The BCIs can provide the experimenter and the patients themselves with real-time feedback independent of motor responses, which makes detecting and assessing number and calculation abilities in patients with DOC possible, as demonstrated in this study. Using a hybrid BCI, we successfully detected number and calculation-related cognitive function in 5 of 11 patients with DOC. Our results showed that for these 5 patients, their number cognition systems (including the IPS and the angular gyrus) were at least partially effective and that their residual brain functions included symbol representation and operation, memory, language and executive function, which are associated with number and calculation cognition.

Mental calculation tasks have been used in rehabilitation training for patients with dementia and stroke. One study reported that 12 patients participated in an experiment involving 6 months of training during which they were asked to perform such tasks as reading and arithmetic for 2 to 6 days per week [58]. Reading aloud and arithmetic calculation was shown to improve frontal function in people with AD-type dementia and could be useful in restoring their communication skills and independence. This improvement may be explained as follows: first, reading aloud and solving arithmetic problems require executive function; and second, in the case of learning therapy, reading aloud and solving arithmetic problems improve general cognitive functions, including communication, independence and conceptualization and executive function. For geriatric patients with confirmed dementia, complex dual-task-based exercise training including motor and backward calculation resulted in improved dual-task performance (deficits in attention-related cognitive performance and measures such as dual-task performance represent early markers of dementia) [59]. Wade et al. used three tasks, including a digit span, to assess the recovery of cognition in patients soon after a stroke [60]. In the present study, of the 11 patients who participated in our experiment, the 5 patients who achieved accuracies that were significantly higher than the chance level improved their levels of consciousness to a large degree (Table 1).

Notably, in our experiment, several patients achieved accuracies (approximately 70 %) that were significantly higher than the chance level but much lower than the performance of healthy subjects (which is generally higher than 90 %, according to our experience). We may explain this result using two key factors. First, because the patients were easily fatigued, we could not collect sufficient training data before each online test block. In fact, we used the data from previous blocks, which were collected on separate days, to update the classifier for the current block; therefore, the performance of the classifier might have been affected. Second, the level of object-selective attention was much lower for patients with DOC than that for healthy subjects. Further studies are required to determine how to improve BCI accuracy for patients with DOC.

Importantly, to perform the experimental tasks, many cognitive functions are required, such as language comprehension (i.e., understanding the task instructions), object selection (i.e., attending to the BCI stimuli), numerical processing (i.e., recognizing the number), and mathematical abilities (i.e., performing the number comparison and mental calculation). The absence of any of these cognitive functions could result in a failure of performance. Furthermore, false-negative findings in BCI studies are possible, even in healthy subjects. Hence, negative results could not be used as evidence for a lack of awareness. However, our positive results did indicate that such cognitive functions existed in these patients, thus demonstrating their residual awareness.

One could argue that patients with DOC often lose the ability to fixate their gaze, which is generally necessary for the visual P300- or SSVEP-based BCIs. Actually, gaze-dependent visual BCIs would be infeasible for a majority of patients with DOC, although the classification performance is generally better for gaze-dependent visual BCIs than for gaze-independent visual BCIs. For patients with DOC, we achieved better results when we employed gaze-independent BCIs. First, auditory-, somatosensory- or MI-based BCI systems may provide an alternative method because these systems are eye gaze-independent approaches [8]. Specifically, auditory BCIs may be suitable for patients whose vision instead of hearing is impaired [61, 62]. For the somatosensory-based BCIs, the tactile stimuli have the advantage of not depending on the auditory or visual system [63]. Several studies have suggested that patients with DOC might be able to use motor imagery to express volitional intent [64]. However, further studies are needed to improve the detection performance of these BCI systems and to facilitate MI training such that they can be used for patients with DOC. Second, various attentional mechanisms, such as covert attention and feature-directed attention, have been investigated to develop gaze-independent visual BCIs. For example, several studies have proposed gaze-independent P300- or SSVEP-based BCIs [65, 66], in which users fixated at the center of the screen and covertly attended to the target stimulus in the visual periphery. In the present study, we used two large visual elements to ease the deployment of covert attention and to improve peripheral visual acuity. We calculated the correlation between the visual function CRS-R scores (Table 1) and the average accuracies (Table 2) for eleven patients. A significant correlation was obtained (*r* = 0.68 and 0.89 based on the visual function CRS-R scores before and after the experiment, respectively, and *p* < 0.05). Furthermore, Tables 1 and 2 show that those patients who achieved significant accuracies also had high visual function scores, including fixation, visual pursuit, object localization and recognition. This finding implies that these visual functions may play an important role in the performance of visual BCIs for these patients.

## Conclusion

As demonstrated in this study, BCIs can help patients with DOC who cannot provide sufficient motor responses to output the results of number recognition and mental calculation. Therefore, BCIs provide an effective tool for the detection of the related abilities. Given our focus on awareness detection, we did not consider the rehabilitation of consciousness in our experimental design. For instance, we did not track the timeliness for patients recovering consciousness. Furthermore, in addition to the BCI trainings, several rehabilitation treatments (e.g., physical and medical rehabilitation treatments) were carried out for each patient in the local hospital. Whether these number- and calculation-based BCI trainings were useful for the patients’ recovery remains unknown. In the future, we will explore the potentials of number- and calculation-based BCI training in the rehabilitation of patients with DOC.

## Abbreviations

DOC: Disorders of consciousness
VS: Vegetative state
MCS: Minimally conscious state
EMCS: Emerged from minimally conscious state
BCI: Brain-computer interface
SSVEP: Steady-state visual evoked potential
GCS: Glasgow coma scale
CRS-R: Coma recovery scale-revised
fMRI: Functional magnetic resonance imaging
EEG: Electroencephalography
TMS: Transcranial magnetic stimulation
IPS: Intraparietal sulcus
AD: Alzheimer’s disease
MoCA: Montreal cognitive assessment
MMSE: Mini-mental state examination
SVM: Support vector machine
ApEn: Approximate entropy
